# Spectroscopic and Kinetic Characterization of Peroxidase-Like π-Cation Radical Pinch-Porphyrin-Iron(III) Reaction Intermediate Models of Peroxidase Enzymes

**DOI:** 10.3390/molecules21070804

**Published:** 2016-06-27

**Authors:** Samuel Hernández Anzaldo, Uriel Arroyo Abad, Armando León García, Daniel Ramírez Rosales, Rafael Zamorano Ulloa, Yasmi Reyes Ortega

**Affiliations:** 1 Centro de Química Instituto de Ciencias, Benemérita Universidad Autónoma de Puebla. Edificio 103H, Ciudad Universitaria, Col. Jardines de San Manuel, Puebla, Pue. 72570, Mexico; samuel.hernandezan@correo.buap.mx (S.H.A.); uri_creek@hotmail.com (U.A.A.); armando_leon2@yahoo.es (A.L.G.); 2 Instituto Politécnico Nacional, ESFM, Ave. Instituto Politécnico Nacional S/N, Edif. 9 U.P. Zacatenco, Col. San Pedro Zacatenco, México City 07738, Mexico; danielesfm@yahoo.com.mx (D.R.R.); davozam@yahoo.com (R.Z.U.)

**Keywords:** pinch-porphyrins, peroxidases models, π-cation radical, spectroscopic studies

## Abstract

The spectroscopic and kinetic characterization of two intermediates from the H_2_O_2_ oxidation of three dimethyl ester [(proto), (meso), (deuteroporphyrinato) (picdien)]Fe(III) complexes ([FePPPic], [FeMPPic] and [FeDPPic], respectively) pinch-porphyrin peroxidase enzyme models, with *s* = 5/2 and 3/2 Fe(III) quantum mixed spin (qms) ground states is described herein. The kinetic study by UV/Vis at λ_max_ = 465 nm showed two different types of kinetics during the oxidation process in the guaiacol test for peroxidases (**1**–**3** + guaiacol + H_2_O_2_ → oxidation guaiacol products). The first intermediate was observed during the first 24 s of the reaction. When the reaction conditions were changed to higher concentration of pinch-porphyrins and hydrogen peroxide only one type of kinetics was observed. Next, the reaction was performed only between pinch-porphyrins-Fe(III) and H_2_O_2_, resulting in only two types of kinetics that were developed during the first 0–4 s. After this time a self-oxidation process was observed. Our hypotheses state that the formation of the π-cation radicals, reaction intermediates of the pinch-porphyrin-Fe(III) family with the ligand picdien [*N*,*N’*-bis-pyridin-2-ylmethyl-propane-1,3-diamine], occurred with unique kinetics that are different from the overall process and was involved in the oxidation pathway. UV-Vis, ^1^H-NMR and ESR spectra confirmed the formation of such intermediates. The results in this paper highlight the link between different spectroscopic techniques that positively depict the kinetic traits of artificial compounds with enzyme-like activity.

## 1. Introduction

Enzymes are proteins that act like natural catalysts, producing a huge increase in the reaction rate by lowering the energetic barrier in the transition state of a process [1,2]. Scientists have tried to mimic this power using synthetic products [2,3]. In this context, peroxidases are among several proteins targeted for replacement by coordination compounds, specifically synthetic metalloporphyrins, due to their similarity to the prosthetic groups found in peroxidases. The catalytic process is performed throughout the oxidation of the transition metal, iron in the case of the metalloporphyrins, and with the same macrocyclic organic ligand. Additionally, magnetic phenomena in iron porphyrins are responsible for the versatile behavior of their chemistry. The spin state of the iron ion is governed by the ligand’s field attachment force and the symmetry of the surrounding ligands [4]. Biologically speaking, the magnetic state of proteins containing the heme group has been recognized as a marker of chemical coordination as well as of subtle biochemical properties [5]. In some biological compounds like ferricytochrome *c*’, horseradish peroxidase and the ferric ion in complexes of tetragonal symmetry, the ground state is composed of the linear combination of two spin states, *s* = 5/2, *s* = 3/2, phenomenon known as quantum mixed spin [6,7]. As well, it has been reported to have a five-coordinated Fe(III)-pinchporphyrins to weak-field anions that were characterized as qms systems which show magnetic properties that span a continuum of magnetic states between the pure *s* = 1/2 and the pure s = 5/2 spin states. Previously, Reed and Guiset [8] proposed a qualitative magnetochemical series based on the iron ligand field deduced from the mixing of *s* = 3/2, 5/2 spin states in porphyriniron(III) compounds. 

**Figure 1:**
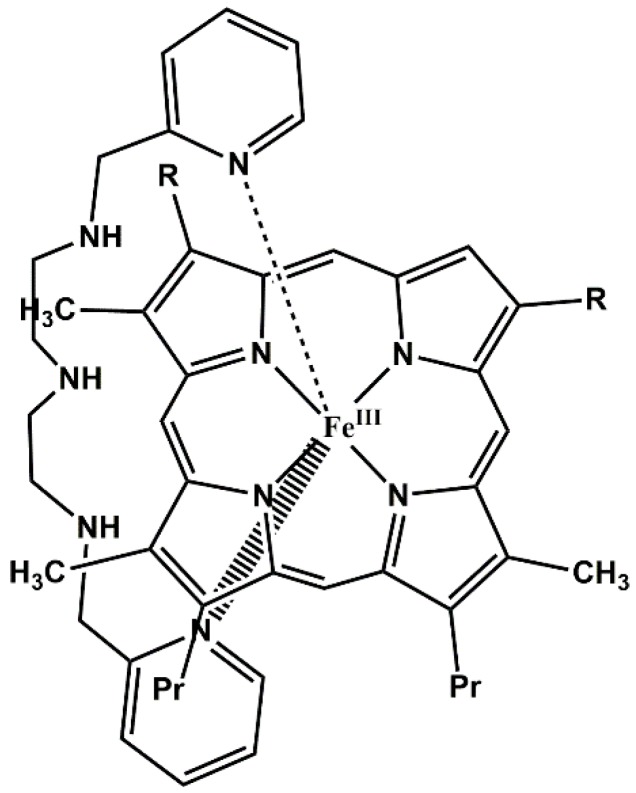
Pinch-porphyrins. When R = –CH=CH_2_ FePPPic; R = –CH_2_–CH_3_ FeMPPic and -H FeDPPic. Pr = methylpropionate.

Peroxidases usually form free radicals that immediately become into dimers that act as substrates or cause oxidation to the species in the system. The rapid reaction rate makes difficult to envision intermediate compounds, however, fine-tuned spectroscopies, like ESR, have being applied due to its sensitivity which allow us to describe the nature of the bond in the complex and even to detect the coupling between species. Previously, we reported a detailed spectroscopy and catalytic studies of a series of pinch-porphyrin-Fe(III) systems, namely [Fe(III)(picdien)(protoporphyrinato dimethyl ester)] = [FePPPic], [Fe(III)(picdien)(mesoporphyrinato dimethyl ester)] = [FeMPPic] and [Fe(III)(picdien)(deuteroporphyrinato dimethyl ester)] = [FeDPPic], although the best kinetic model of peroxidases is yet to be reported (Figure 1). The pinch-ligand significantly alters the iron environment in these complexes, producing physical characteristics of qms of the Fe(III) which correlate with catalytic activity [3]. Overall, it is clear that the chain size of the pinch-axial ligands on the structure, the qms of Fe(III), and the ratio of the mixtures of *s* = 3/2, 5/2 spin states have a correlation to the catalytic activity as peroxidases. One of the most difficult challenges in the catalytic mechanism of peroxidases has been the demonstration of the existence of its intermediates, compounds I and II. These intermediates have a ferryl Fe(IV)=O at high spin and free radical. Besides, the unpaired spins of the ion Fe(IV) are coupled with the radical, giving a total spin of 3/2, which allowed us to see the intermediate by an ESR signal with g ~ 4. In this paper we report *in situ* spectroscopic studies using UV-Vis, ^1^H-NMR and ESR and the description of the catalytic steps in the formation of compounds I and II.

## 2. Results and Discussion

Almost 40 years ago, Peters and Dundford rose the question about the mechanism of compound I formation [9]. Although it was thought to be a reversible substrate complex, the final conclusion was that the formation of the intermediate mostly involves an irreversible, 2-electron equivalent oxidation of the enzyme; nevertheless, some authors would disagree due to the emergence of the location in the oxidizing equivalents which may be different in different enzymes [10]. As well, there is plenty of information that a reversible enzyme–substrate complex (termed compound 0) is involved in the pathway towards the complete oxidation of the peroxidases although its structure has remained controversial [11].

**Figure 2:**
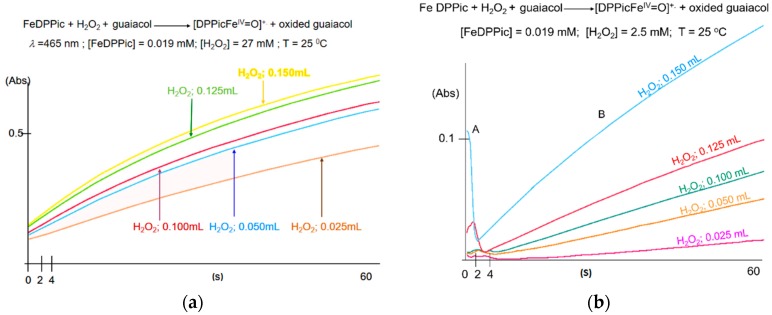
(**a**) Single kinetics showing only the guaiacol oxidation products; (**b**) Two types of kinetics, oxidation of **A** pinch-porphyrin and **B** guaiacol, or three in some cases, were observed when 2.5 mM [H_2_O_2_] was added, one magnitude order lower than for activity catalytic studies.

The kinetic study by UV-Vis swept at 500 nm of **1**–**3** (Figure 2) shows that under these conditions the limiting step in their oxidation is the formation of compound I, since the first signal at ~1 s is rapidly transformed into the next one belonging to compound II at ~3 s. As we previously discussed, **1**, **2** and **3** are oxidized by themselves and as seen in Figure 2, after the formation of compound II the absorption disappears after 4 s. Additionally, a saturation is seen when the ideal concentration is surpassed ≈10 times and the UV-Vis swept is not fast enough to detect the formation of either compound I or II.

**Figure 3:**
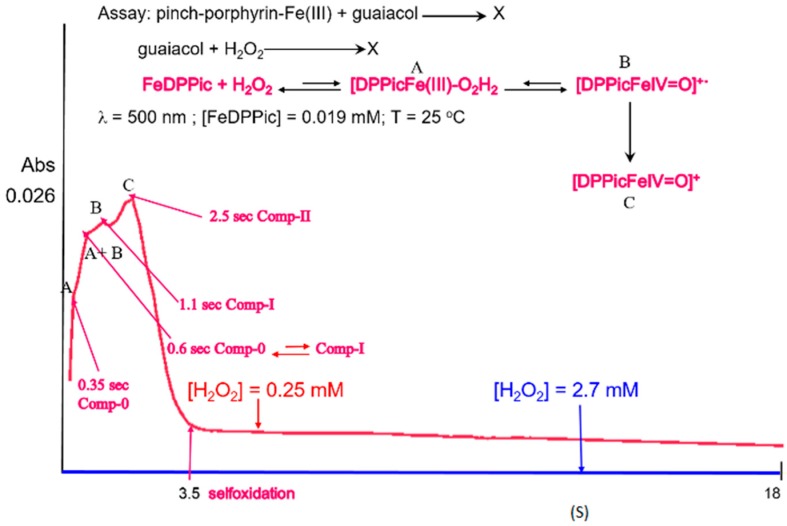
Kinetic pathway of the reaction [FeDPPic]_(MeOH)_ + H_2_O_2(aq)_ at the first four seconds of reaction and at 500 nm (―). The blue line (―) corresponds to the condition with the H_2_O_2(aq)_ concentration one magnitude order higher.

When the guaiacol tests were performed to establish the conditions in which the pinch-iron porphyrin acts at its best as a peroxidase (new-pinchporphyrin complexes with quantum mixed spin ground state *s* = 5/2, 3/2 of iron (III) and their catalytic activity as peroxidase [12]), two kinetic curves were observed: **A** and **B**. In these experiments the concentrations of pinch porphyrin-Fe(III) and guaiacol were kept constant and the assays were performed in a 123–156 mM range of aqueous hydrogen peroxide concentrations. The kinetic curve **A** was seen in the first 4 s of the reaction and after this time the products of guaiacol oxidation formed curve **B** (Figure 2a,b). Having established the approximate concentrations of [FePPPic], [FeMPPic] and [FeDPPic] where they show the two kinetic curves, we proceeded to assess the optimum concentrations of peroxide to observe only the kinetics **A**. The conditions were as follow: 0.0123 mM for [FePPPic], 0.134 mM for [FeMPPic] and 0.25 mM for [FeDPPic] (Figure 3), noticing that they showed very similar kinetic behavior. In the case of [FePPPic] the formation of compounds I and II was so rapid that it was only possible to observe the disappearance step of the pinch porphyrin. Many different conditions were tried to carry out this reaction and was still not possible to see the formation of the intermediates. The reaction mixtures in these assays only contained the pinch porphyrin-Fe(III) compounds and H_2_O_2_, therefore, the formed curves could only correspond to the formation of the compounds I (ferryl π-cation radical) and compound II (ferryl π-cation), the intermediates of the catalytic peroxidase enzymes. In each test performed, it was also possible to observe a color change in the solutions from green to red corresponding to the depletion of **A** and the formation of **B**, as shown in the catalytic activity test of horseradish peroxidase. All assays were performed at λ = 500 nm and 298 K. 

Figure 3 shows four reaction kinetic curves of the model compounds. At 0.35 s the reaction rate is extremely fast and probably correspond to the [P-Fe(III)-O_2_H_2_] complex, followed by a change and a curve with a steeper slope until 0.6 s time. Later, at 0.5 s the reaction follows slower kinetics, and finally, the slope is newly more positive informing of an increasing in the reaction rate. In Figure 3 we have suggested what step of the mechanism corresponds to each kinetic curve. 

**Scheme 1:**
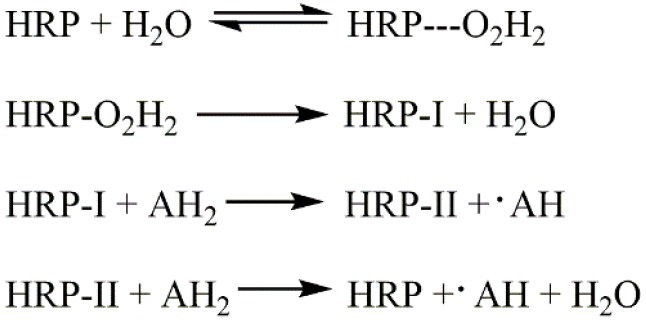
The normal peroxidase cycle. HRP Horseradish peroxidase, HRP---O_2_H_2_ Compound 0, HRP-I compound-I, HRP-II compound-II.

Peroxidase activity in biological systems was first reported in the 19th century [13] and a reaction mechanism was proposed for these enzymes (Scheme 1).

It has been shown that compound 0 is formed quickly but the complicated O-O cleavage is the limiting step of the peroxidase cycle [14]. This is a major contribution for the decrease of the rate in the second kinetic trace. After this second step, compound II is produced quickly as the guaiacol oxidation is carried out. The mechanism matches perfectly with the kinetic steps of the pinch-porphyrin oxidation process as well as for its stages.

### 3.1. UV-Vis 

**Figure 4:**
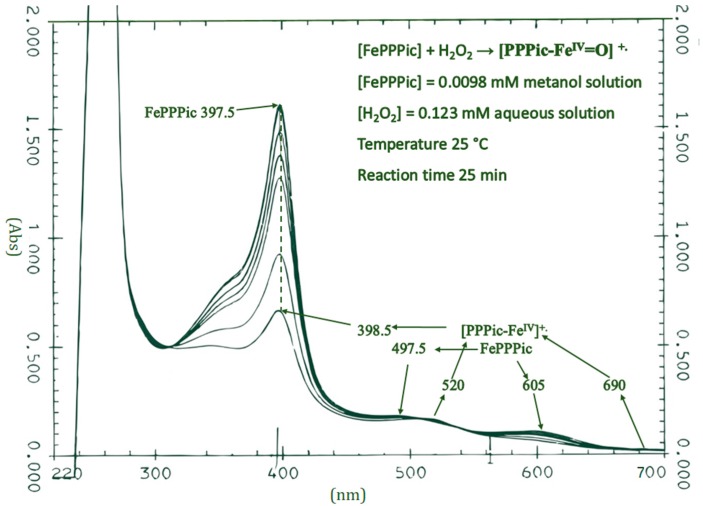
Snapshots of [FePPPic]**^+.^** formation observed for 25 min. Soret bands decrease indicating that the [FePPPic] is being auto-oxidized because of the absence of guaiacol in the reaction media, similar to HRP-I (compound I of horseradish peroxidase) behavior in an excess of hydrogen peroxide, which reduces compound I. *d*-*d* transitions energetically change showing a new spectrum.

The UV-Vis spectra of **1**–**3** showed notable changes compared with the parent compounds. The Soret band decreased until quenched when the pinch-porphyrins auto-oxidized because there was no guaiacol in the reaction medium. Electronic *d*-*d* transitions were shifted from 487.5 nm for [FePPPic] to 520 nm and from 605 nm to 690 nm for [PPPicFe^IV^=O]^+·^ (Figure 4), from 480 nm for [FeMPPic] to 522 nm for [MPPicFe^IV^=O]^+·^ (Supplementary Materials Figure S1), and for [FeDPPic] *d-d* band from 476 nm to 520 nm, from 587 nm to 690 nm for [DPPicFe^IV^=O]^+·^ (Supplementary Materials Figure S2). A change in color from green to red is observed. The UV-Vis studies showed that for this spectroscopy the very rapid pinch-porphyrin oxidation did not provide an opportunity to observe the formation of compound I and later the signal of compound II, due to a higher rate of its transformation; however, kinetic and ESR studies showed signals typical of compound I.

UV-Vis spectral data of compound I and compound II for native peroxidase enzymes or their model compounds, have shown considerable differences [15,16,17].

In Table 1 already reported UV-Vis data are shown for selected native peroxidases and model compounds, and the UV-Vis data for [PP-Fe^IV^=O]. It is clear that the *d*-*d* transition λ_max_ values are in accordance with other reports [7,17,18,19,20,21,22,23,24,25]. It was not possible to do the same with the Soret bands because there is a destruction of the pinch-porphyrin rings in the pinch-porphyrin-I and II, which proved that these reaction intermediates are very reactive.

### 3.2. ^1^H-NMR of Compound I

**Figure 5:**
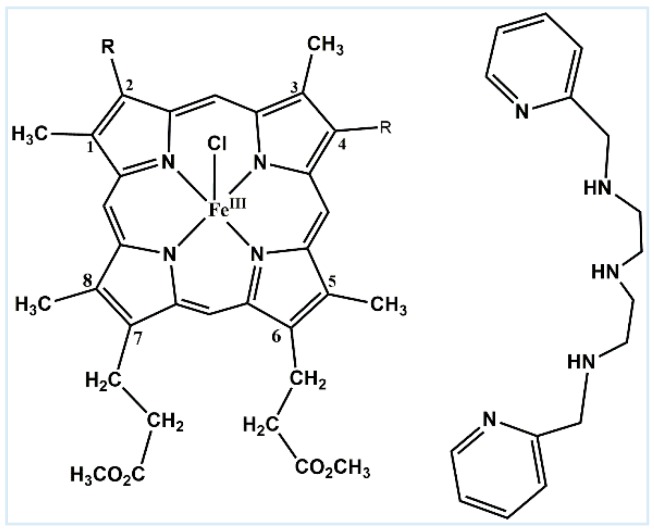
Precursors of the Fe(III)-dimethyl ester porphyrin and 1,9-bis-(2-piridyl)-2,5,8-triazanonane (Picdien). Protoporphyrin R = –CH=CH_2_, Mesoporphyrin R = –CH_2_–CH_3_, Deuteroporphyrin-H.

Previously we reported the ^1^H-NMR analysis assignment for the pinch-porphyrin complexes Figure 4 [3] according to Figure 5.

**Figure 6:**
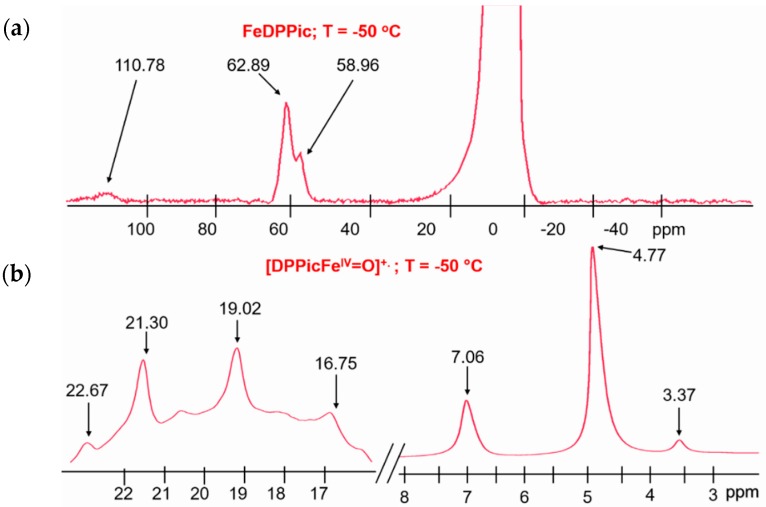
(**a**) ^1^H-NMR of [FeDPPic] with chemical shifts of *meso*-protons at 110.78 ppm and methyl-protons at 63.89 and 58.96 ppm; (**b**) ^1^H-NMR of [DPPicFe^IV^=O]^+·^ with the highest chemical shifts at 22.67 ppm and with no possibilities of assignation.

Overall, the relaxation times values (T1) for Fe(III)-pinch porphiryns (parent compounds) are visible as downfield-shifted signals, and they are usually assigned according to their intensity (from x to *s* y). The signals with the highest intensity and the best definition belong mostly to the CH_3_ groups of the heme, which are more distant of the paramagnetic center with shorter relaxation times. These methyl groups show isotropic shifts at higher frequencies by the strong electronegativity coming from the Fe(III) ion. This electronegativity is so high that it strongly attracts both pi and sigma electrons of the methyl groups, compressing their electron density. The signals of medium intensity are due to the Hα of the vinyl and propionate moieties of the heme and to the Hβ protons of the proximal picdien (Figure 6) [26,27,28].

The formation of the ferryl radical and the oxidation of Fe^3+^ → Fe^4+^ caused by H_2_O_2_ were confirmed by the ^1^H-NMR spectra under low temperature conditions that allow detecting such species. These data show the appearance of a new set of hyperfine-shifted resonances that are characteristic of Fe^4+^ ferriheme-like complexes, replacing the ones in the Fe^3+^ species of [FeDPPic] (Figure 6), [FeMPPic] (Supplementary Materials Figure S3) and [FePPPic] (Supplementary Materials Figure S4) [3].

The chemical shift displacement of the protons in the heme group is explained by the loss of *s* = 5/2 in the quantum mixed spin character. Now that the Fe^3+^ ion has been substituted with Fe^4+^ with a *d^4^* configuration, which is coupled with the spin radical giving a *s*_total_ = 3/2, this results in the decreasing of the sensitivity towards *s* = 3/2 and *s* = 5/2 contributions.

The positions of chemical shifts in the ^1^H-NMR [pinch-porphyrins Fe^IV^=O]^+·^ spectra inform us that the spin state of iron ion is lower than for the pinch-porphyrin parent compounds since the chemical shift responds to δα(*s* + 1) [28,29]. The iron (IV) ion becomes more electronegative for the higher oxidation state and its bond with the nitrogen atom of pyridine proximal is shorter. The ion position respect to the porphyrin-ring decreases its electron-withdrawing effect on sigma and pi electrons. The ^1^H-NMR spectra for the [pinch porphyrins-Fe^IV^=O]^+·^ correspond to a new magnetic structure.

The ^1^H-NMR studies of this pinch-porphyrin pi-cationic-radical, [PFe^IV^=O]^+·^ family, show that when there is no guaiacol in the reaction medium, the reaction between [PFe^IV^=O]^+·^ and H_2_O_2_ produces a new set of highly reactive compounds which immediately destroy the porphyrinic structures. The pinch porphyrin pi-cationic-radicals show lower spin states than the iron ion bond just to the pinch porphyrins, and this idea comes from the ^1^H-NMR and ESR magnetic studies. These reaction products show characteristics of the oxidation intermediate compounds of peroxidase enzymes, compounds I and II (Table 2).

### 3.3. ESR Spectroscopy

**Figure 7:**
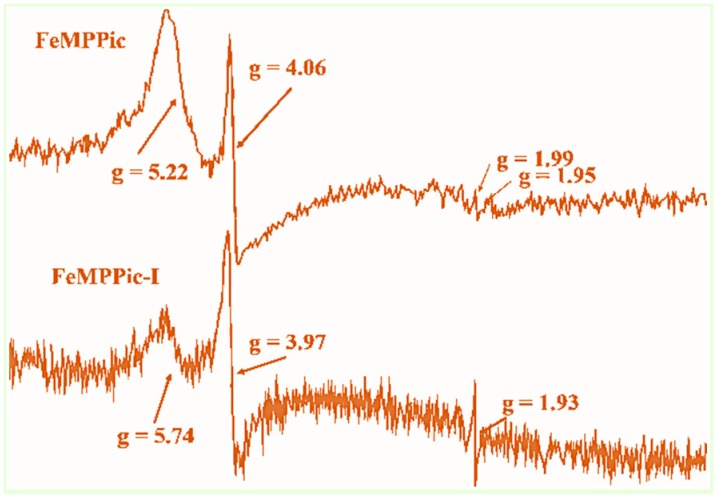
Top: ESR spectrum of [FeMPPic] at 77 K in methanol solution with two species of Fe(III) with qms; *s* = 3/2, *S* = 5/2, species with g = 5.22, 1.99 contains more *s* = 5/2 in qms; species with g = 4.06, 1.95, contains more *s* = 3/2 in qms. Bottom: [FeMPPic]-I with two species of iron ion, one with g = 5.74 and higher percentage of this species in qms but in less proportion than the second species (g = 3.97) with *s* = 3/2. At g = 1.93 the radical signal is present.

The ESR spectra of [PPicFe^IV^=O]^+^ clearly showed the transformation of the quantum mixed phenomena spin species to the Fe^4+^. The picdien axial ligand has shown the unexplained ability of its coordination to these porphyrins, to produce only two ligand-field Fe^+3^ environments in the complexes, these being *s* = 3/2 and *s* = 5/2. To further characterize these species, many repeated ESR experiments were carried out in our laboratory showing that the signals correspond to the qms for the intermediates compounds [FePPPic]-I, [FeMPPic]-I and [FeDPPic]-I [3] (Figure 7).

Moreover, the two paramagnetic species *s* = 3/2, *s* = 5/2 are stabilized by the picdien ligand axially coordinated to the three different pinch porphyrins, this would correspond to the coexistence of structures of Fe(III) ion very small out-of-plane position and the out-of-plane position, respectively. The qms species with higher proportion of *s* = 5/2 decrease when the Fe^4+^ is formed; hence, the spectral line shape changes to give the free radical a unique signal at g = 1.93. The radical signal is near of the signal with g = 2 corresponding to two axial ESR spectra for the three model intermediates of compound I (Figure 5). The signal at g ~ 4, corresponding to the *s* = 3/2 increases and its line shape is very similar in the three pinchporphyrins radical cationic ferryl (Table 3) [32].

**Figure 8:**
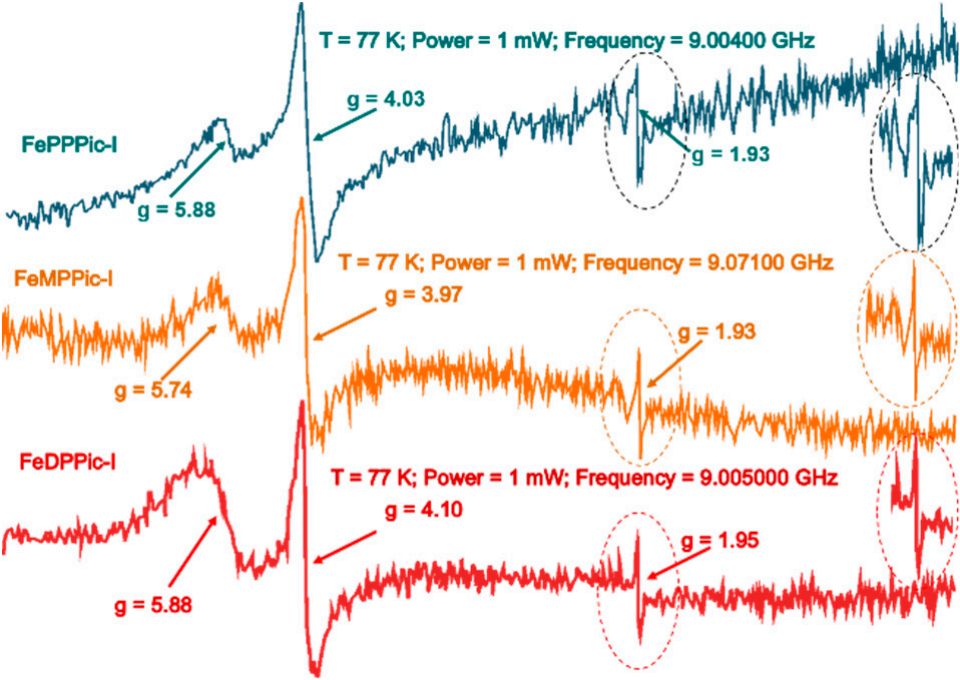
ESR axial spectra of [PPicdienFe^IV^=O]^+·^ with species of iron ion at higher energy with qms (g > 5.7), proportion decrease from [FePPPic]-I > [FeMPPic]-I > [FeDPPic]-I. Species with *s* = 3/2 (g ~ 4) and with the highest proportion in each sample. The *s* = 3/2 corresponds to three unpaired spins, which stem of the Fe(IV) ion low spin (*s* = 1) ferromagnetically coupled with radical with *s* = 1/2, giving a total spin of *s* = 3/2.

Figure 8 displays the ESR spectra of compound-I from [PPPicFe^IV^=O]^+·^, [MPPicFe^IV^=O]^+·^ and [DPPicFe^IV^=O]^+·^. These spectra present two species of iron ion with qms, *s* = 3/2, *s* = 5/2, and in major proportion the *s* = 5/2 species (g values of 5.74, 5.88). Other species only with *s* = 3/2 (g values ~ 4, 1.9), this last signal is typical of a radical.

ESR spectra of FePPic-I show a radical signal different to the reported for HRP-I [35,36,37,38,39,40]; although, it is possible that these signals contain the one at g ~ 2 for axial spectra of qms species and the axial spectra with *s* = 3/2. It is clear that each ESR spectrum is a snapshot of some of the changes in the oxidation state of the iron ion. When we finished each FePPic-I spectrum, we tried to obtain the following one, then the signal was quenched.

### 3.4. Reaction Mechanism 

**Scheme 2:**
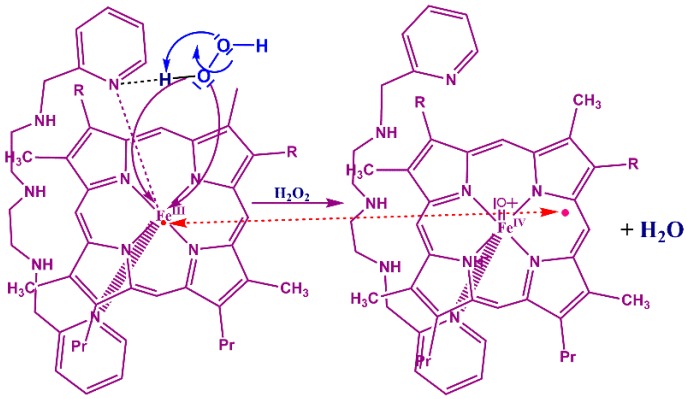
The intermediate compound 0 formed faster if the Fe (III) has less stability to receive less electronic contribution of its porphyrin macroligand.

**Figure 9:**
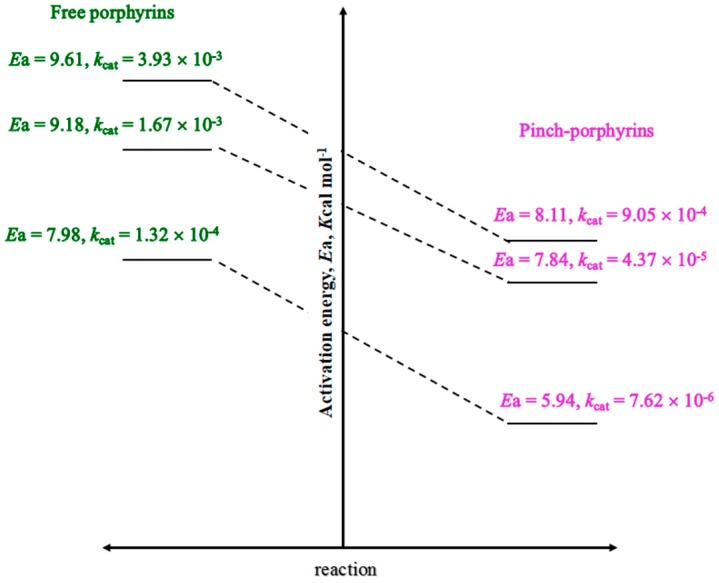
Comparison of the activation energies from three compounds with and without the pinch-axial-ligand.

Our proposed reaction mechanism is shown in Scheme 2 [3] starting from compound 0 to produce compounds I and II, which are obtained more or less quickly depending on the stability of compound 0. Although, the activation energies to produce compound 0, quantified in other work [40], were smaller for [FePPPic] (5.4 Kcal·mol^−1^) < [FeMPPic] (7.84 Kcal mol^−1^) < [FeDPPic] (8.11 Kcal mol^−1^) (Figure 9). These values of *E*_act_ inform us that Fe(III) ion in the [FePPPic] is more electronegative than in [FeMPPic] and [FeDPPic], which is consistent with the electron-withdrawing vinyl groups that are present in positions 2, 4 of the porphyrin ring. The ethyl and vinyl groups present in [FeMPPic] and [FeDPPic] are electron-donors and decrease the Fe(III) electronegativity.

## 3. Material and Methods

The complexes [1,9-bis(2-pyridyl)-2,5,8-triazanonane]-(protoporphyrinato)iron(III) [FePPPic], -(mesoporphyrinato)-iron(III) [FeMPPic] and -(deuteroporphyrinato)iron(III) [FeDPPic]], were synthesized starting from the parent compounds chloro-(porphyrinato)iron(III), -(mesoporphyrinato)iron(III) and -(deuteroporphyrinato)iron(III) and 1,9-bis(2-pyridyl)-2,5,8-triazanonane (pic) [3]. Electronic spectra were measured at 298 K on a UV-3100 spectrophotometer (Shimadzu, Kyoto, Japan) in methanol solution of pinch-porphyrins in the range 200–800 nm, Table 4 summarizes these conditions. The microwave X-band (9.4 GHz) of a JEOL JES-RES3X spectrometer (Jeol Co. Tokyo, Japan), power of 1 mW, field amplitude 0–500 mT, and frequency of: 9.00400 GHz for [[FePPPic]], 9.07100 GHz for [FeMPPic] and 9.005000 GHz for [FeDPPic]. NMR measurements were carried out on a JEOL Eclipse 300 spectrometer (Jeol Co. Tokyo, Japan) at 213 K, frequency 300 MHz, pulse width 6.7 s, No. repetitions 128, FT size 131072, total acquisition time 5 min, field amplitude of −80 to +80 ppm, acquisition time 1.422 s [FePPPic], 0.800 s [FeMPPic] and 0.800 s [FeDPPic].

The pinch-porphyrins and hydrogen peroxide concentrations for ESR and NMR-^1^H studies in methanolic solutions were the same that for UV-Vis studies.

## 4. Conclusions

More information to explain the always interesting mechanism of the intermediates resulting from the oxidation process of the peroxidases enzymes is been added. Three new pinch-porphyrin π-radical cationic products, which are formed by the oxidation process of enzyme-like synthetic compounds, were observed and characterized by UV-Vis, ^1^H-NMR and ESR spectroscopies. It is important to note that a unique half lifetime is seen in the present intermediates, a characteristic that allowed us to perform all the analyses. As well, their kinetic behaviors are consistent with previous results reported for possible intermediate model compounds proposed to be responsible for catalytic activity of peroxidases. Importantly, a correlation between the ratios of their qms properties with the catalytic activity was established by the integration of these data. It is clear that the limiting step in the oxidation process is the formation of compound 0, being the heterolytic cleavage the determinant factor in the balance of the overall pathway. 

## Figures and Tables

**Table 1:** Selected λ_max_ from different sources of compound I.

Compound Sources	λ_max_ (nm)	Reference
Horseradish Peroxidase-I	475, 530	[7]
Horseradish Peroxidase-I	529, 555	[18]
Cytochrome P450-I	515, 572	[19]
Cytosolic Heme Binding Protein-I	540, 547	[20]
Heme-thiolate Peroxygenase-I	538, 571	[21]
Hemoglobin I (*from Lucina pectinate)-*I	519, 650	[22]
Chloro(2-oxa-3-oxotetramesitylporphinato)Fe(III)	556, 601	[23]
Chloro(2-oxa-3-oxotetrakis(2,6-dichlorophenyl)porphinato)Fe(III)	560, 610	[24]
[*meso*-Tetrakis(2,4,6-trimethyl-3-sulfonatophenyl)-porphinato]Fe(III)	526, 580	[25]
[FePPPic]-I	520, 690	This work
[FeMPPic]-I	522, -	This work
[FeDPPic]-I	520, 690	This work

**Table 2:** Selected ESR spectra data of compound I and compound II of peroxidases and their model compounds, contrasting the chemical shifts for [pinch porphyrinsFe^IV^=O]^+·^ presented herein.

Compound Source	Chemical Shift (ppm)	Reference
Horseradish Peroxidase-I	80, 7	[30]
H42A-Horseradish Peroxidase-I	80, 6.5	[31]
Cytochrome c peroxidase-I	35, 5	[32]
Chloroperoxidase-I	48, 8	[33]
Chloro(2-oxa-3-oxotetramesitylporphinato)Fe(III)	20, 7.6	[34]
Chloro(2-oxa-3-oxotetrakis(2,6-dichlorophenyl)-porphinato)Fe(III)	20, 8.9	[34]
(py)tetra-*m*-tolyporphyrin)Fe(III)-I	−38, −17.7	[28]
Manganese Peroxidase-I	40, 7.7	[35]
Lignin peroxidase-I	42, 7.4	[35]
[FePPPic]-I	39, 7.12	This work
[FeMPPic]-I	29, 7.02	This work
[FeDPPic]-I	19, 7.06	This work

**Table 3:** ESR spectra data of compound I and compound II intermediates of peroxidases and peroxidase models, contrasting the chemical shifts for [pinch porphyrinsFe^IV^=O]^+·^ presented in this work.

Compound Source	g Values	Reference
Horseradish Peroxidase-I	6.38, 5.38, 2.00	[35]
Pea ascorbate peroxidase-I	6.04, 5.27, 1.99	[36]
Chloro(2-oxa-3-oxotetramesitylporphinato)Fe(III)	3.98, 2.00	[33]
Chloro(2-oxa-3-oxotetrakis(2,6-dichlorophenyl)-porphinato)Fe(III)	3.72, 1.99	[33]
4,6-di(*tert*-Butyl)-2-aminophenolCu(II)-I	2.32, 2.06	[37]
2-Anilino-4,6-di(*tert*-butyl)-2-aminophenolCu(II)-I	2.27, 2.07	[37]
Oxyferrous hp450scc-DHC-I	2.44, 2.25, 1.91	[38]
Lignin peroxidase-I	6.6, 1.95	[39]
[FePPPic]-I	5.88, 4.03, 1.93	This work
[FeMPPic]-I	5.74, 3.98, 1.93	This work
[FeDPPic]-I	5.88, 4.10, 1.95	This work

**Table 4:** Conditions of Compound I and II formation, starting of pinchporphyrins by spectroscopy of UV-Vis at 25 °C, ^1^H-NMR at −50 °C and ESR at 77 K.

[Pinch-Porphyrin]	ca [Pinch-Porpyrin]*_f_*, Methanol, mM	ca [H_2_O_2_]_f_, Aqua, mM
[FePPPic]	0.0098	0.123
[FeMPPic]	0.0487	1.14
[FeDPPic]	0.057	1.34

## Supplementary Materials

Supplementary materials can be accessed at: http://www.mdpi.com/1420-3049/21/7/804/s1.
