# Supplementary material for: Spectroscopic and Kinetic Characterization of Peroxidase-Like π-Cation Radical Pinch-Porphyrin-Iron(III) Reaction Intermediate Models of Peroxidase Enzymes
Source: Molecules. 2016 Jun 27;21(7):804. doi: 10.3390/molecules21070804 (PMC6273987; doi:10.3390/molecules21070804)
Supplement: Supplementary file 1 [file molecules-21-00804-s001.pdf]

Supplementary Materials: Spectroscopic and Kinetic Characterization of Peroxidase-Like π-Cation Radical Pinch-Porphyrin-Iron(III) Reaction Intermediates, Models of Peroxidases Enzymes

Samuel Hernández Anzaldo, Uriel Arroyo Abad, Armando León García, Daniel Ramírez Rosales, Rafael Zamorano Ulloa and Yasmi Reyes Ortega

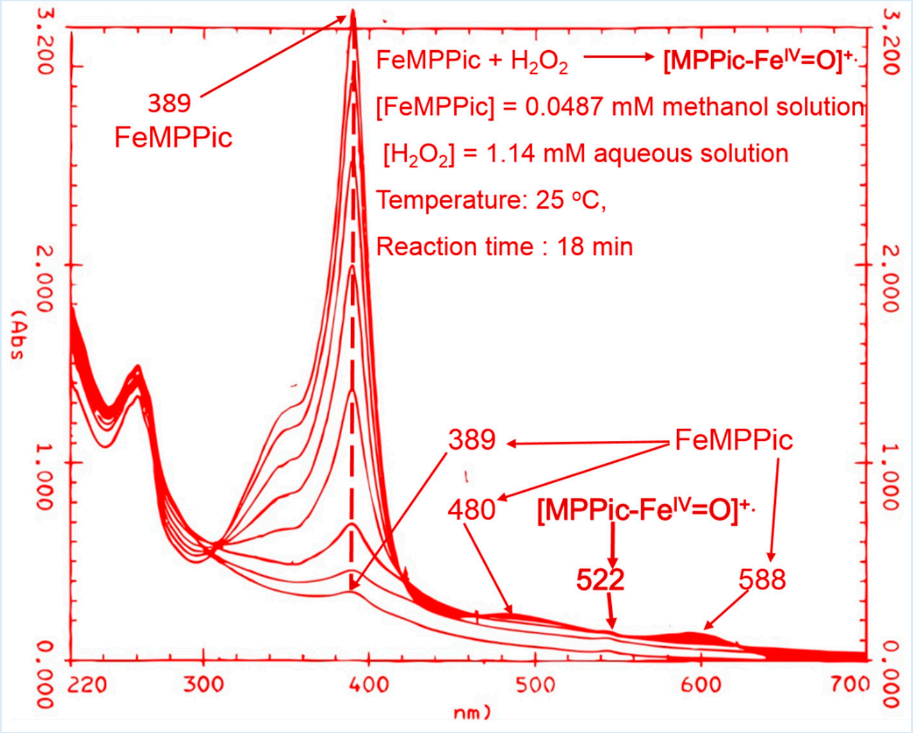

Figure S1. Snapshots of [FeMPPic]$^{+\cdot}$ formation observed 18 min. Soret band decreases indicating that the FeMPPic is autooxidizing because there is not guaiacol in reaction mixture, similar to HRP-I (compound I of Horseradish peroxidase) behavior in an excess of hydrogen peroxide, which reduce compound I. Two *d-d* transitions of the parent compound change and only is possible to observe the transition at 522 nm, corresponding to FeMPPic-I.

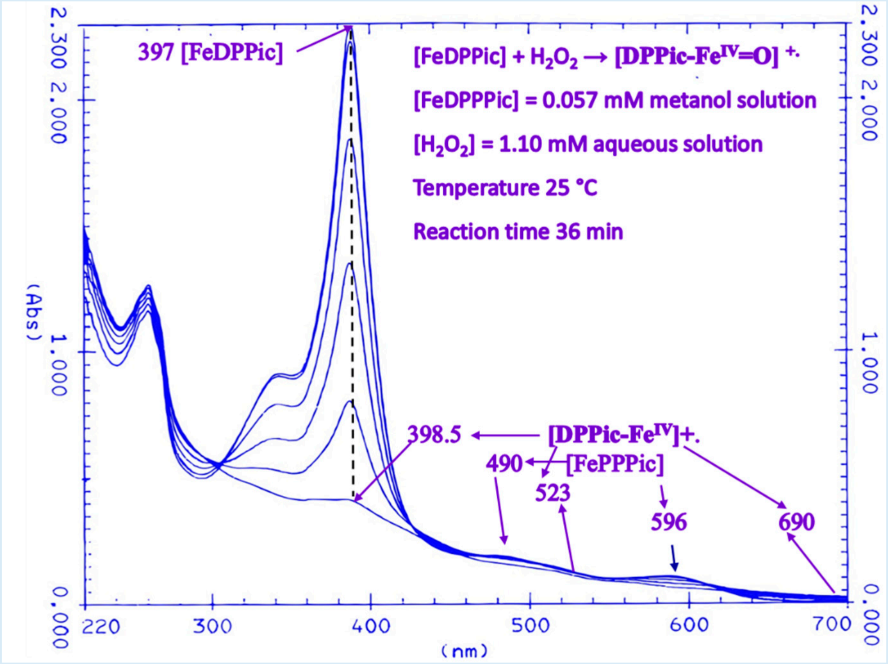

Figure S2. Snapshots of $[FeDPPic]^{+\cdot}$ formation observed 13 min. Soret band decreases indicating that the FeMPPic is autooxidizing because there is not guaiacol in reaction mixture, similar to HRP-I (compound I of Horseradish peroxidase) behavior in an excess of hydrogen peroxide, which reduce compound I. Only one *d-d* transitions at λ_{max} = 522 nm is observed, which corresponds to FeDPPic-I.

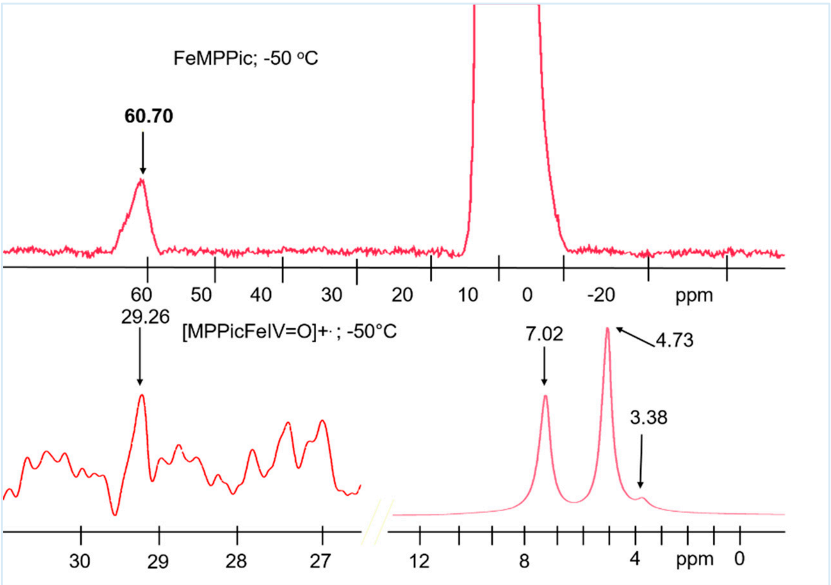

Figure S3. ^{1}H-NMR spectrum for (top) FeMPPic with chemical shifts at 60.70 ppm; (bottom) $[MPPicFe^{IV}{=}O]^{+\cdot}$ with signals shifted at 29.26 ppm.

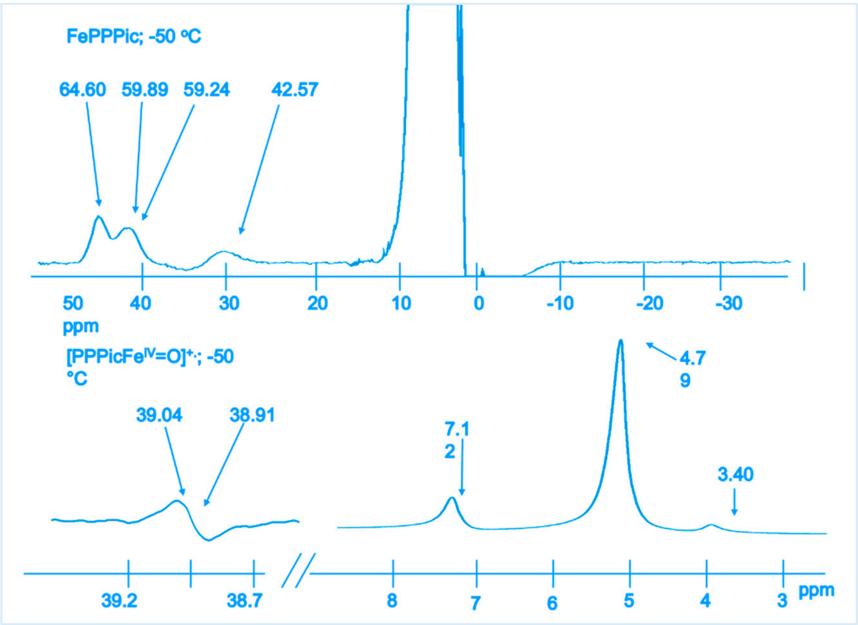

Figure S4. ^{1}H-NMR spectrum for (top) FePPPic with chemical shifts at 64.60 ppm; (bottom) [PPPicFe^{IV}=O]$^{+·}$ with signals shifted at 39.04 ppm.

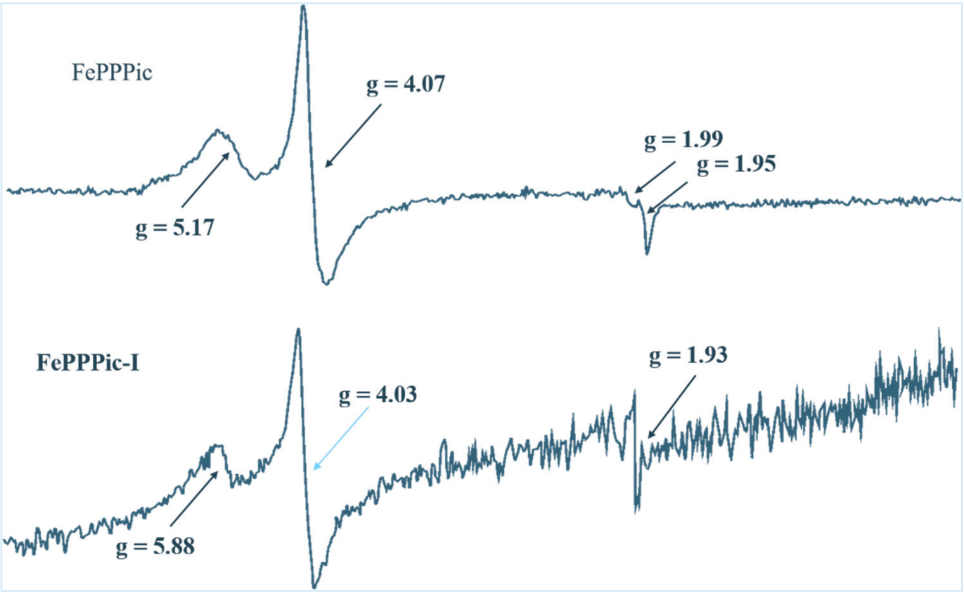

Figure S5. ESR spectrum for (top) FePPPic with signals of two qms species of Fe(III); (bottom) [PPPicFe^{IV}=O]$^{+·}$ with signals of one species of Fe(III) with qms, other species of Fe(IV) with g value of 4.3 and a signal characteristic of radical at g = 1.93.

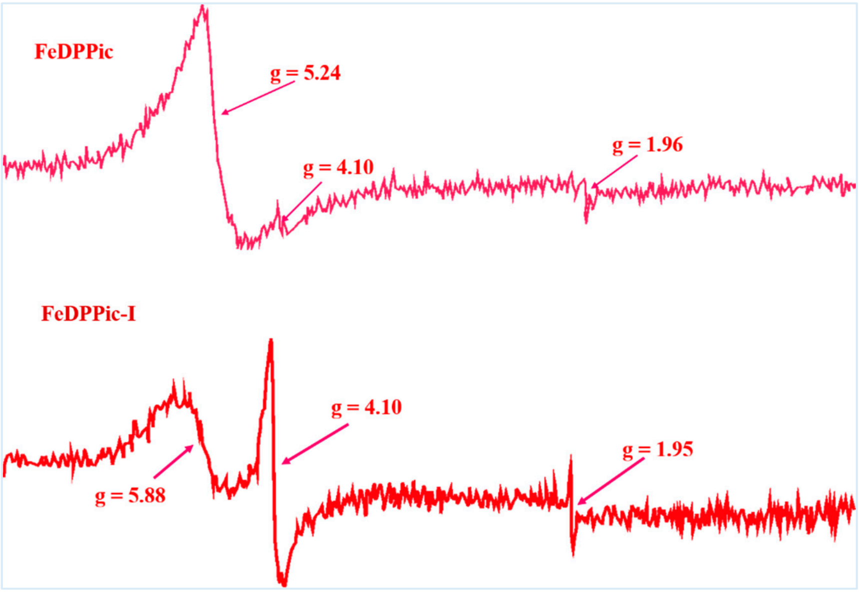

Figure S6. ESR spectrum for (top) FeDPPic with signals of two qms species of Fe(III); (bottom) $[PPicFe^{IV}{=}O]^{+\cdot}$ with signals of one species with Fe(IV) and g value of 4.3, and other signal of radical with g value of 1.95.
